# Chirality and the angular momentum of light

**DOI:** 10.1098/rsta.2015.0433

**Published:** 2017-02-28

**Authors:** Robert P. Cameron, Jörg B. Götte, Stephen M. Barnett, Alison M. Yao

**Affiliations:** 1 School of Physics and Astronomy, University of Glasgow, Glasgow G12 8QQ, UK; 2 Department of Physics, University of Strathclyde, Glasgow G4 0NG, UK

**Keywords:** chirality, optical angular momentum, molecules, physical chemistry

## Abstract

Chirality is exhibited by objects that cannot be rotated into their mirror images. It is far from obvious that this has anything to do with the angular momentum of light, which owes its existence to rotational symmetries. There is nevertheless a subtle connection between chirality and the angular momentum of light. We demonstrate this connection and, in particular, its significance in the context of chiral light–matter interactions.

This article is part of the themed issue ‘Optical orbital angular momentum’.

## 1. Introduction

The word chiral was introduced by Kelvin to refer to any geometrical figure or group of points that cannot be brought into coincidence with its mirror image, thus possessing a sense of handedness [1]. It derives, in fact, from the Greek for hand: 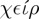 (E. Eleftheriadou 2015, private communication). In the language of point group theory, a chiral form is devoid of improper rotational symmetry elements [2]. The word dissymmetry was used by Pasteur to convey this more negative perspective [3,4]. Barron effectively extended Kelvin’s definition of chirality to include time in addition to space by distinguishing between ‘true’ and ‘false’ chirality, the former being exhibited by systems that can exist in two distinct enantiomeric (enantiomorphic) states interconvertible, up to a proper rotation, by a parity inversion but not by a time reversal [5].

**Figure 1.**
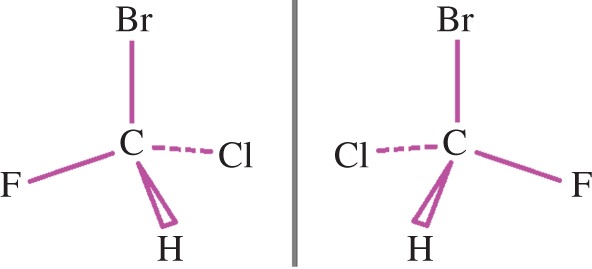
The opposite mirror-image forms or *enantiomers* of bromochlorofluoromethane, a prototypical handed or *chiral* molecule. (Online version in colour.)

Chirality pervades the natural world, from the enigmatic preferences of fundamental forces [6,7] to the helices traced out by the arms of galaxies [8] and even the plates of *Stegosaurus* [9,10]. In particular, many molecules can enjoy a seemingly stable existence in either a left- or a right-handed form which are distinct mirror images of each other, as was established by the pioneering works of Pasteur [11], van ’t Hoff [12] and Le Bel [13]. These opposite enantiomers often interact rather differently with living things, as chirality is also inherent to life: amino acids, sugars and other biomolecules besides are chiral and their chirality is crucial to their biological function [14]. To give but one of many striking examples, one enantiomer of methamphetamine is recognized as being a harmful narcotic, whereas the other enantiomer is relatively harmless, being employed, in fact, as a decongestant. The two enantiomers of a simple chiral molecule are depicted in figure 1.

One of the principal means by which we are able to probe and harness molecular chirality is through the use of light that is itself chiral. Circularly polarized light in particular, which was discovered by Fresnel, is a truly chiral influence [2,15]. The electric- and magnetic-field vectors trace out helices in space [16], which can be left- or right-handed. Well-established chiral light–matter interactions include optical rotation, i.e. the differential *refraction* of left- and right-handed circularly polarized light [17,18], circular dichroism, i.e. the differential *absorption* [19–22], and Raman optical activity, i.e. the differential Raman *scattering* [22–25]. Many other chiral electromagnetic interactions exist, of course [2,26–32].

That light can carry a well-defined angular momentum was recognized by Poynting [33], who inferred by analogy with a rotating cylindrical shaft that a beam of circularly polarized light carries an intrinsic angular momentum parallel to the direction of propagation. The existence of this so-called spin was confirmed by Beth [34]. In the modern understanding, the spin is 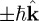 per circularly polarized plane-wave mode photon of wavevector **k** [35], where the upper and lower signs refer, respectively, to left- and right-handed circular polarization in the optics convention [36] and a circumflex indicates a unit vector.

As the spin of a beam of circularly polarized light differs for left- and right-handed circular polarizations, it is natural, perhaps, to enquire as to its connection with the chirality of light and, moreover, to ask whether spin plays any explicit role in chiral light–matter interactions like those described above. It turns out, however, that there is no profound relationship between chirality and spin. One may appreciate this simply by noting that a parity inversion of the beam reverses the handedness of the beam (and the direction of propagation) while, nevertheless, leaving the spin unchanged. Spin derives not from the screw sense of the helices but instead from the sense of rotation of the field vectors: the spin can be cast as an integral over 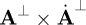, for example, with **A**^⊥^ the solenoidal magnetic vector potential and × denoting the conventional vector product. This resembles the angular momentum 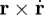 associated with the rotation of a particle’s position vector **r**, say.

At first glance, then, it might appear that chirality and the angular momentum of light are disparate subjects: chirality is the concept of handedness while the angular momentum of light, in particular spin, is associated with rotation rather than any form of inversion. Developments in recent years have revealed, however, that these two fields are, in fact, subtly intertwined. The purpose of this short paper is to elucidate and consolidate some of the advances in this direction.

In what follows, we consider ourselves to be in an inertial frame of reference, adopting a right-handed Cartesian coordinate system *x*,*y*,*z* with time *t*. Indices taken from the start of the Roman alphabet (*a*,*b*,*c*,…) may take on the values *x*, *y* or *z* and a double appearance of an index implies summation over *x*, *y* and *z*. We take a microscopic view, focusing upon freely propagating light or the interaction of this light with individual molecules. We work within the semiclassical domain, where the electromagnetic field is treated classically and everything else is treated quantum mechanically [37].

## 2. The angular momentum of light

It is now well established that light carries spin and also orbital angular momentum [38–41]. Less widely appreciated at present, however, is the fact that these are but two of many angular momenta carried by light, in the sense that there are many rotational symmetries inherent to Maxwell’s equations. In this section, we give a brief overview of the basic description in fundamental electromagnetic theory of the angular momentum of light, in particular, those facets of it that may not be familiar to the reader but nevertheless take centre stage in the context of the chiral light–matter interactions discussed in the following section. We work here in a system of units with *ϵ*_0_=*μ*_0_=*c*=1.

There has been much controversy in the past over what constitutes a ‘true’ angular momentum. We argue that an angular momentum is, fundamentally, a property of a system with the dimensions of an angular momentum, the conservation of which is associated with a rotational symmetry according to Noether’s theorem [42–46]. An angular momentum in this sense does not necessarily have a corresponding quantity in quantum mechanics that satisfies the usual commutation relation nor does it need to be a pseudovector.^1^

### (a) Manifestly intrinsic angular momenta

Freely propagating light is rather special in that it possesses, in particular, an infinite number of manifestly intrinsic angular momenta. The existence of these is intimately associated with the massless and vectorial character of the electromagnetic field [45,47].

**Figure 2.**
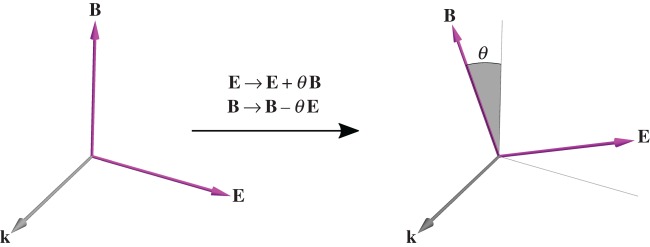
The conservation of helicity, a manifestly intrinsic angular momentum distinct from spin, is associated with the fact that the electric and magnetic field vectors can be rotated about the direction of propagation. (Online version in colour.)

At the heart of this collection is the helicity [48],
2.1
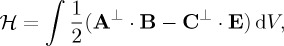
with 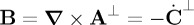 the magnetic field, **C**^⊥^ the solenodial electric pseudovector potential and 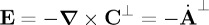 the electric field [36,49–51]. Here and elsewhere ⋅ denotes the scalar product. The gauge-invariant potentials **A**^⊥^ and in particular **C**^⊥^, although less familiar than **E** and **B** perhaps, nevertheless make natural appearances here and in what follows. Their significance is discussed in more detail in [51], for example. Helicity 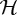 takes on a value equivalent to 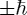 per circularly polarized plane-wave mode photon, in line with the concept of helicity familiar from particle physics. The conservation of helicity is associated with a rotational symmetry [52] which in infinitesimal form is
2.2
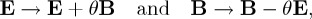
with *θ* an infinitesimal Lorentz pseudoscalar angle. This sees the electric and magnetic field vectors of each plane-wave mode comprising the electromagnetic field rotated about the wavevector **k** of the mode through *θ* [47,53], as depicted in figure 2. The existence of this symmetry embodies the idea of electric–magnetic democracy [54]: the fact that the electric and magnetic fields reside on equal footing in the strict absence of charge [45,46,50,55,56]. Looking at the integrand of 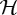, we identify a helicity density
2.3
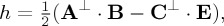
which has interesting properties, not least the fact that it is time-independent for monochromatic light.

**Figure 3.**
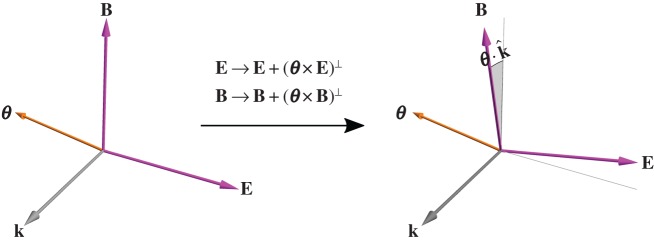
The conservation of spin, a manifestly intrinsic angular momentum distinct from helicity, is associated with the fact that the electric and magnetic field vectors can be rotated about any direction in space (provided this is done in a manner that leaves them perpendicular to the direction of propagation and each other, of course). (Online version in colour.)

The more familiar spin [50,57]
2.4
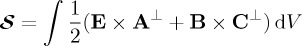
in contrast takes on a value equivalent to 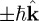 per photon [35], as described above. The conservation of spin is associated with a rotational symmetry which in infinitesimal form is [50,58,59]
2.5
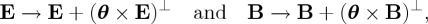
with ***θ*** a pseudovector of infinitesimal magnitude. This sees the electric and magnetic field vectors of each plane-wave mode rotated through an angle 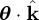 [50,58,59], as depicted in figure 3.

The *ab*-infra-zilch [45,47], so named because of its connection with Lipkin’s *ab*-zilch^2^ described below, is a component of a rank-two rotational pseudotensor, given by
2.6
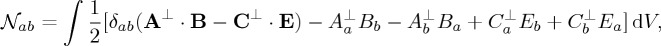
which takes on a value equivalent to 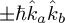 per photon, where 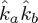 denotes a dyadic or outer product. The conservation of the *ab*-infra-zilch is associated with a rotational symmetry which in infinitesimal form sees the electric and magnetic field vectors of each plane-wave mode rotated through an angle 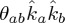, with *θ*_*ab*_=*θ*_*ba*_ an infinitesimal angle.

It seems that this pattern extends in the obvious way: in general, there exists an angular momentum with components that take on values equivalent to 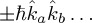 per photon and which is associated with a rotational symmetry that sees the electric and magnetic field vectors of each plane-wave mode rotated through an angle 
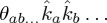
 [61]. Let us emphasize that helicity, spin, the *ab*-infra-zilches and so on are *distinct* from each other. Although the helicity, the *z* component of spin, the *zz*-infra-zilch and so on take on similar values for the particular case of a beam of light propagating in the +*z* direction, say, the differences in these angular momenta become clear when one considers more exotic forms of light [62]. A subtlety worth noting is that helicity, spin, the ab-infra-zilches and so on are *not* synonymous with the concept of polarization, although their values certainly *depend* upon polarization. The distinction may be appreciated simply by noting that horizontal and vertical polarizations are *distinct* and yet are associated with the *same*, vanishing helicity, for example.

Interesting analogies can be drawn between these angular momenta and other, more familiar, quantities in physics. The continuity equation
2.7
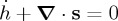
for helicity [53] is reminiscent of the familiar continuity equation
2.8
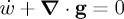
for energy, with *w*=(**E**⋅**E**+**B**⋅**B**)/2 the usual energy density and **g**=**E**×**B** the usual energy flux density or linear momentum density, that is, Poynting’s vector [63]. The continuity equation
2.9
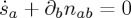
for spin [47], with *n*_*ab*_ the integrand of 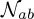, is reminiscent of the familiar continuity equation
2.10
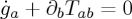
for linear momentum, with *T*_*ab*_ the components of the usual stress tensor [36]. Note that **s** plays a dual role in that it is both a helicity flux density and a spin density [53], much as **g** plays a dual role in that it is both an energy flux density and a linear momentum density [63]. A further analogy can be drawn between helicity and electric charge, both of which are signed, conserved quantities with no sense of orientation [47]. Spin is then analogous to electric current and simple optical fields can be constructed that are reminiscent of various types of current-carrying wire [47].

### (b) Extrinsic and complete angular momentum

This theme issue celebrates the discovery by Allen *et al.* [38] that a beam of light with helical phase fronts of the form 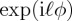 possesses a well-defined orbital angular momentum parallel to the direction of propagation equal, in essence, to 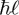 per photon. This discovery marked the start of a lively field of research [39,40,64–66] in which the role of the angular momentum of light in its various guises has been investigated for ever more complicated realizations, reaching from light with fractional orbital angular momentum mean [67–70] to vector vortex beams that combine both spin and orbital angular momentum in a spatially inseparable manner [71,72]. For such beams neither the orbital nor the total angular momentum mean is necessarily an integer multiple of 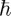, which is why it is important to unravel the individual contributions and their connection to chirality.

The (exact) orbital angular momentum is [50,57]
2.11
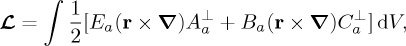
with the characteristic dependence upon helical phase fronts deriving from the presence of **r**×**∇**, the *z* component of which is 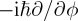 in cylindrical coordinates: this gives 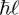 when acting on 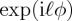. The orbital angular momentum 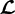 is extrinsic in that **r** makes an explicit appearance. One component of 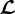, that parallel to the total linear momentum 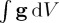, is nevertheless independent of the choice of location of the origin and can thus be said to be quasi-intrinsic [73–76]. The conservation of orbital angular momentum is associated with a rotational symmetry which in infinitesimal form is [50]
2.12
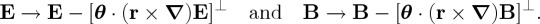
This can be interpreted as the closest approximation to a rotation of the spatial distribution of the electromagnetic field, leaving the orientations of the electric- and magnetic-field vectors unchanged, which is consistent with the requirement that the electric and magnetic fields be solenoidal [50]. Note that the combination of the spin and orbital rotational symmetries (2.5) and (2.12) gives a complete geometrical rotation of the electromagnetic field, associated with the conservation of the complete angular momentum [36,43]
2.13
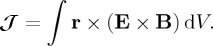
It is natural, perhaps, to ask whether there is a quantity related to orbital angular momentum as helicity is related to spin [61]. It appears, however, that it is not possible to construct a non-vanishing quantity of this sort. This may be appreciated by noting that the integrand of 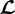 is perpendicular to the wavevector **k** at each point in reciprocal space [57,77,78] so that the component of this integrand along **k** is zero, which is reminiscent of the fact that the projection of a particle’s orbital angular momentum parallel to the particle’s direction of motion is zero, as 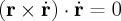.

### (c) Boost angular momenta

Our attention has been focused thus far on the familiar concept that rotations (of the usual, circular character) in space are connected to the conservation of angular momenta. Somewhat less familiar but equally important is the fact that light also carries angular momenta for which the conservation is associated instead with ‘rotations’ of hyperbolic character in space–*time*.

The boost angular momentum
2.14
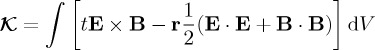
sits on equal footing with 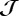 in as much as the two appear together as parts of a more basic object: the angular momentum *tensor* [36,79]. The conservation of boost angular momentum implies that the centre of energy of the electromagnetic field moves with constant velocity [57,79] and is associated with a boost rotational symmetry transformation which in infinitesimal form is [79]
2.15
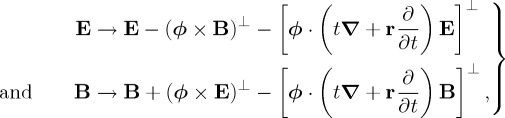
with ***ϕ*** a rapidity vector of infinitesimal magnitude. It does not appear possible to separate 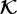 into non-vanishing spin and orbital contributions [79].

It turns out that there are, in fact, an infinite number of boost rotational symmetry transformations which are closely analogous to those underpinning the conservation of helicity, spin, the *ab*-infra-zilches and so on [45]. First
2.16
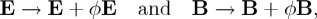
with *ϕ* an infinitesimal rapidity angle, is the boost analogue of the helicity symmetry transformation (2.2) and sees the amplitude of each plane wave comprising the electromagnetic field modulated by a factor of (1+*ϕ*) [45]. Next
2.17
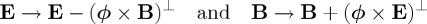
is the boost analogue of the spin symmetry transformation (2.5), and sees the amplitude of each plane wave modified by a factor 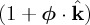: this is nothing but the first contribution to (2.15). It turns out, however, that the conservation laws associated with these symmetry transformations are rather trivial. This can be attributed to the oscillatory nature of electromagnetic waves [61].

We note finally here the existence of
2.18
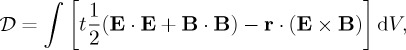
which might be referred to as the boost helicity, as its relation to boost angular momentum is analogous to that between helicity and the complete angular momentum [61]. The conservation of boost helicity can be interpreted as a reflection of the dispersion relation *ω*=*c*|**k**| for freely propagating light [45,61] or indeed the existence of a symmetry transformation which in infinitesimal form is [43]
2.19
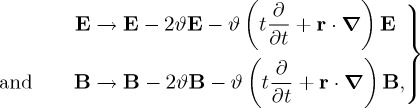
with *ϑ* an infinitesimal rapidity angle. The first terms here modify the amplitudes of the waves comprising the electromagnetic field and the second terms modify their wavelengths, while maintaining the speed of light. This embodies the scale invariance of Maxwell’s equations [45,61]. It is interesting to note that the sum of the symmetry transformations (2.16) and (2.19), with *ϕ*=*ϑ* now, is simply a boost with rapidity *ϕ*=*ϑ* of each plane-wave mode comprising the electromagnetic field antiparallel to the wavevector **k** of the mode.

### (d) A possible source of confusion

Let us conclude the present section now by highlighting a subtle feature of freely propagating light which may be a source of confusion. Maxwell’s equations as written in the strict absence of charge [36],
2.20
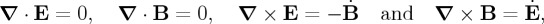
exhibit a kind of self-similarity [45,47] in that various integrals of the electric and magnetic fields satisfy Maxwell-like equations; for example,
2.21
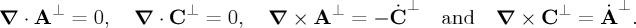
Similarly for various derivatives of the electric and magnetic fields; for example,
2.22
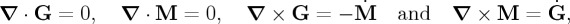
with 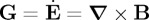 and 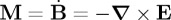, say. It follows immediately that each of the angular momenta described above has an infinite number of higher and lower extensions [45,47], as do other quantities, including energy and linear momentum. If we consider the helicity, for example, and replace, superficially, the fields with their time derivatives, we obtain
2.23
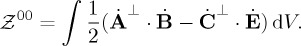
This is the 00-zilch [48,60,80], which was discovered before the helicity, in fact, and takes on a value equivalent to 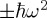 per photon, with the *ω*^2^ here deriving from the product of two time derivatives. That the 00-zilch is conserved follows from the Maxwell-like equations (2.22) in the same way that the conservation of helicity follows from Maxwell’s equations themselves (2.20) [45,47]. The 00-zilch is not an angular momentum. Its conservation is associated with a symmetry which in infinitesimal form is [45,52,81]
2.24
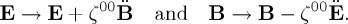
This resembles a duality rotation, but differs crucially via the appearance of the second-order derivatives and fails to qualify as a rotation, in as much as *ζ*^00^ does not have the dimensions of an angle. Indeed, 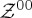 itself does not have the dimensions of an angular momentum. Looking at the integrand of the 00-zilch, we identify
2.25
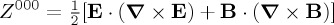
as a 00-zilch density. For strictly monochromatic light of angular frequency *ω* [82–85],
2.26
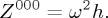
It is for this reason perhaps that the 00-zilch has been *mistaken* for helicity. In general, however, there is no simple relationship between *Z*^000^ and *h*, and the two must not be regarded as being synonymous [47].

The results described above hold for light propagating freely in the strict absence of charge. Even the presence of bound charges in an overall neutral medium sets stringent conditions on the conservation of helicity at a material interface [86,87]. In the presence of unpaired charges, the quantities described above are not by themselves conserved [51]. Nevertheless, it is possible to formulate rigorous continuity equations that include source terms explicitly [88].

## 3. Chiral light–matter interactions

In this section, we explain that some of the angular momenta described above, in particular, the helicity and related quantities, arise naturally in the context of certain chiral light–matter interactions. We work here in the international system of units, in which the constants *ϵ*_0_, *μ*_0_ and *c* appear explicitly to give expressions that can be directly compared with experimental measurements.

Unlike spin, helicity is faithful to the chirality of light. Indeed, the total helicity 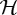 is a time-even (Lorentz) pseudoscalar and hence a measure of ‘true’ chirality [5]. The mirror-image forms of an electromagnetic field have equal and oppositely signed values of the helicity, irrespective of their orientations. This should be clear physically as, in a mirror, each left-handed circularly polarized plane wave (helicity 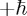 per photon) comprising the field is instead right-handed (helicity 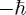 per photon) and vice versa [53]. Similar characteristics are exhibited by the total 00-zilch 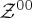, for example, although it is noteworthy that this and the other higher- and lower-order extensions of 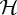 are not invariant under full rotations in space–time, specifically boosts. Indeed, 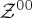 is but one component of a rank-two pseudotensor [60].

Let us emphasize before proceeding that chirality and helicity are *not synonymous*. Chirality is, according to the original definition [1] and its natural extensions [5], a *concept* of general importance, whereas helicity is instead an *angular momentum*, particular to light. It is meaningless to ask how much ‘chirality’ is located within a given region of space, or how ‘chirality’ flows through an optical field, for example. Such questions can be asked of helicity, however, even though densities and flux densities are, as entities by themselves, not unique; a reflection in turn of the freedom available in choosing a Lagrangian density for the electromagnetic field or indeed a gauge [89]. A particular helicity density *h* and related quantities can be identified in simple calculations pertaining to certain chiral light–matter interactions and, in this sense at least, chirality and chiral light–matter interactions are seen to be connected with the angular momentum of light. It is most appropriate, perhaps, to regard *h* and related quantities as *chirality functions* for the electromagnetic field in this context [2,85,90].

Let us focus our attention explicitly here upon a particular chiral light–matter interaction and some of its manifestations. Suppose first then that a small, non-magnetic, chiral molecule is held fixed notionally in weak, monochromatic, far-off-resonance light that is otherwise freely propagating and to which the molecule has been introduced adiabatically, with the angular frequency *ω* of the light somewhere in the visible or near-infrared region, say. The light simply drives oscillations in the charge and current distributions of the molecule, in particular inducing an electric-dipole moment, electric-quadrupole moment components and a mechanical magnetic-dipole moment given to the leading order of present interest by [2,37]
3.1
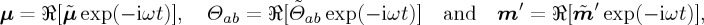
respectively, with
3.2
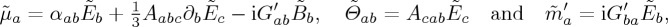
where the *α*_*ab*_ are dispersive electric dipole–electric dipole polarizability components, the *A*_*a*,*bc*_ are dispersive electric dipole–electric quadrupole polarizability components, the *G*′_*ab*_ are dispersive electric dipole–magnetic dipole polarizability components and
3.3
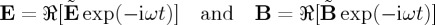
are the electric and magnetic fields, respectively, of the illuminating light, which satisfy the free-field Maxwell equations (2.20). The rotational degrees of freedom of the molecule can be accounted for rigorously by including rotational states in the polarizabilities or heuristically by working with oriented forms and performing appropriate rotational averages at the end of calculations. The double appearance of *G*′_*ab*_ here can be understood in terms of electric–magnetic democracy and energy conservation [91].

### (a) Optical rotation

There is a certain energy associated with the oscillations driven in the charge and current distributions of the molecule by the light [61,92,93], the cycle-averaged, rotationally averaged form of which is
3.4
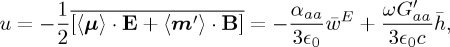
with an overline indicating a cycle average, angular brackets indicating a rotational average, 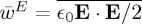 a cycle-averaged electric energy density and 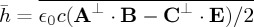 a cycle-averaged helicity density. The quantity *G*′_*aa*_/3 appearing here has opposite signs for opposite molecular enantiomers. The interaction energy *u* thus differs for opposite molecular enantiomers illuminated by light with non-vanishing helicity as embodied by 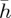.

It can be shown in a rigorous quantum mechanical calculation [61] that the phenomenon of natural optical rotation [2,17,18] derives from this basic interaction energy. The key elements of the argument can be outlined as follows. Consider a weak, circularly polarized plane wave propagating through a simple model of a fluid with *N* molecules per unit volume. We assume this medium to be essentially homogeneous but nevertheless sufficiently dilute that interactions between the molecules are of negligible importance. The total energy associated with the oscillations driven in the charge and current distributions of the molecules as the light propagates through the medium is essentially
3.5
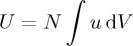
and the phase refractive index of the medium follows in turn as
3.6
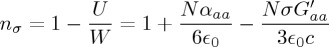
to first order in *N* and the polarizabilities, with *σ*=±1 for left- and right-handed circular polarizations and
3.7
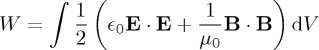
the energy of the light in the dilute limit. The deviation in phase speed away from *c* is thus dictated by the ratio of the interaction energy *U* to the energy *W* of the light. Importantly, *U* and hence *n*_*σ*_ differs for opposite circular polarizations due to the presence of the helicity and hence *σ*. This circular birefringence gives rise in turn to natural optical rotation, the characteristics of which resemble those of a duality rotation: the rotational symmetry transformation that underpins the conservation of helicity, as seen in (2.2). Explicitly, the angle of optical rotation suffered by linearly polarized light upon traversing a physical path length *l* follows from basic geometrical arguments [2] and the above as
3.8
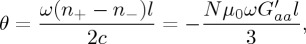
to leading order, which is the well-established result [2,18]. The picture just outlined is equivalent, of course, to other pictures, including those based upon light forward scattering [2]. The identification of a chirally sensitive interaction energy as the basis of the phenomenon nevertheless reveals a number of new possibilities for chiral light–matter interactions. We highlight two of these novel manifestations of optical activity below.

### (b) Chiral optical force

The basic interaction energy *u* described above as the basis of optical rotation varies in general with the position of the molecule in the light. The molecule thus experiences a force due to the light, the cycle-averaged, rotationally averaged form of which is essentially [92,93]
3.9
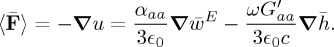
The first term is the familiar dipole optical force, which acts to accelerate the molecule in a manner governed by electric energy gradients in the light; the same dipole optical force used to trap atoms in optical lattices and which underpins the operation of optical tweezers [94–96], for example. The second term is new and acts to accelerate opposite molecular enantiomers in opposite directions, in a manner governed by helicity gradients in the light. This is our discriminatory optical force for chiral molecules [92,93]. Its form was also recognized, independently, by a number of other authors in their considerations of an isotropic chiral dipole of unspecified constitution [97–99].

It is possible to conceive of light sporting helicity fringes [62] for which 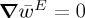 everywhere, while 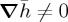 in general, so that the resulting force 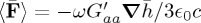 is absolutely discriminatory to leading order; pointing in opposite directions for opposite enantiomers [92,93].

**Figure 4.**
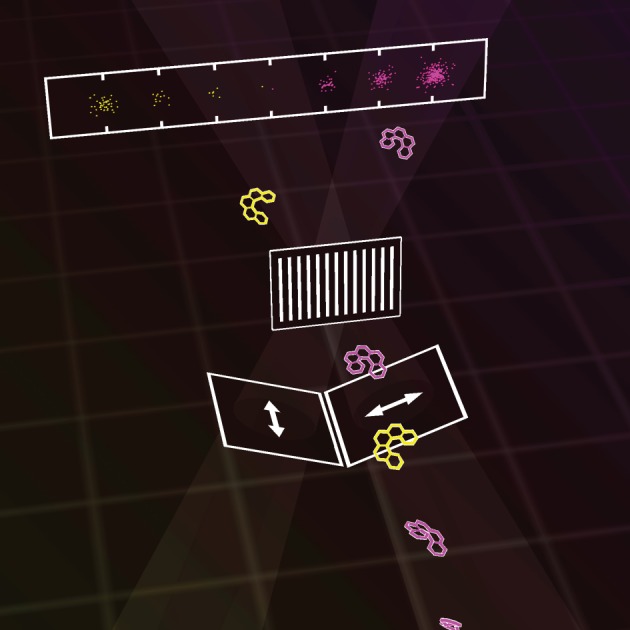
A discriminatory chiral diffraction grating diffracts left-handed molecular matter waves to the left, say, while diffracting right-handed waves to the right instead. Figure courtesy of Cameron *et al*. [93]. (Online version in colour.)

This could form the basis of a number of new devices for chiral molecules, including a chiral Stern–Gerlach deflector capable of spatially separating opposite molecular enantiomers in the Newtonian regime [92,93], a chiral diffraction grating capable of diffracting chiral molecular matter waves in the de Broglie regime while nevertheless treating left- and right-handed forms equally [92,93], and a discriminatory chiral diffraction grating which combines elements of the aforementioned devices [93], as depicted in figure 4.

### (c) Chiral rotational spectroscopy

The basic interaction energy described above as the basis of optical rotation and our chiral optical force affects molecules in different rotational states differently and might be observed, therefore, via the rotational spectrum of such molecules. If the chirally sensitive contribution to the interaction energy is
3.10
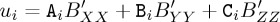
for a particular initial rotational state and
3.11
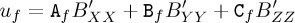
instead for a final rotational state, where *B*_*XX*_=−*G*_*XX*_′/*ω*+(*A*_*Y*,*ZX*_−*A*_*Z*,*XY*_)/3, etc., are molecule-fixed combinations of the optical activity polarizability components and A_*f*_≠A_*i*_, B_*f*_≠B_*i*_ and C_*f*_≠C_*i*_ in general, then the associated energy difference between the states is
3.12
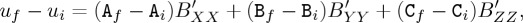
which can be observed as part of a light-induced shift in the rotational resonance frequency of such molecules. This chiral rotational spectroscopy promises a number of exciting capabilities, including the ability to determine oriented chiroptical information, determine enantiomeric excess, probe the molecular chirality of racemates, detect and characterize isotopic molecular chirality, and distinguish well and in a chirally sensitive manner between even slightly different molecular forms [100].

### (d) Rayleigh optical activity

The oscillating charge and current distributions driven in the molecule by the light are themselves a source of electromagnetic radiation. This Rayleigh scattered light is largely suppressed in a suitably uniform medium [2] but can be quite significant in rarified samples, in particular in the gas phase. Consider, then, a collection of molecules clustered around the origin while being randomly oriented and illuminated by light as before. Adopting a sum over independent scatterers approach, as is appropriate for the gas phase, it is found that the intensity of Rayleigh scattering light seen at position **R** in the far field is essentially [101]
3.13
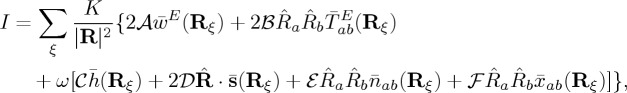
with 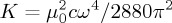 and **R**_*ξ*_ the position of the *ξ*th molecule. It is assumed here that the direction of observation 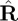 is not parallel to the direction of propagation of any of the plane waves comprising the illuminating light. Both 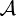 and 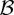 take the same values for opposite molecular enantiomers and are thus insensitive to the chirality of the molecules, while 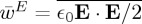 is a cycle-averaged electric energy density and the 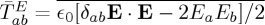 are cycle-averaged electric linear momentum flux density components. By contrast, 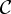, 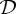, 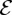 and 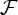 each have equal magnitudes but *opposite signs* for opposite molecular enantiomers and so *are* sensitive to the chirality of the molecules, while 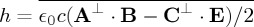 is a cycle-averaged helicity density, 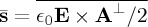 is a cycle-averaged spin density and the 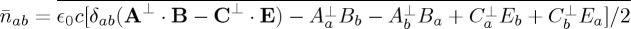 are cycle-averaged *ab*-infra-zilch densities.^3^ Explicitly,
3.14
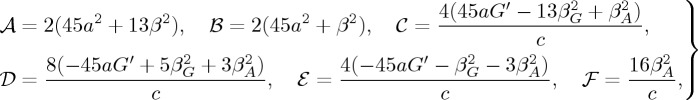
with
3.15
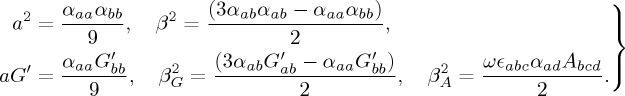
This result for *I* can be applied to a single circularly polarized plane wave illuminating the molecules, in which case it reduces to the usual form [2,23,24], as it should. It can also be applied to more exotic forms of illuminating light, which opens the door to new possibilities [101]: superchiral light enables an enhancement analogous to that recently demonstrated for luminescence-detected circular dichroism [90,102,103]; *σ*–*σ* light enables the removal of unwanted, achiral background contributions to the scattered light [101] which have thus far plagued attempts to observe Rayleigh optical activity by traditional means [2]; lin ⊥ lin light, which is by itself essentially achiral, enables the extraction of chirally sensitive information when coupled with the direction of observation, in a manner that avoids spurious contributions due to circular dichroism [101]. A challenge facing such approaches is that molecules must be confined appropriately to subwavelength regions.

### (e) Other chiral light–matter interactions

We have shown that natural optical rotation, our chiral optical force, chiral rotational spectroscopy and natural Rayleigh optical activity are all related to each other and to the angular momentum of light, in particular the helicity and related quantities. Many more chiral light–matter interactions exist, of course. For some of these, the angular momentum of light again makes explicit appearances. Circular dichroism [19–22], for example, has been tied to the 00-zilch (or equivalently in this context, helicity) and to superchiral light [90,102,103]. For others, there is no obvious connection with the angular momentum of light. This seems to be true in particular for nonlinear interactions where chiral sensitivity comes in at electric-dipole order, so that the magnetic field in particular is of little importance and the appearance of a quantity like helicity would therefore be surprising.

## 4. Discussion

We have considered chirality, the angular momentum of light and some of the connections between these fields of research.

Many questions remain, of course. An obvious one, perhaps, is whether the more familiar orbital angular momentum can be said to play a role in chiral light–matter interactions. Light carrying helical phase fronts is manifestly chiral, with the phase fronts screwing to the left for ℓ>0 and to the right for ℓ<0. It seems, however, that this is not the case for small chiral molecules. Typically, the twist inherent to the wavefronts occurs over a spatial extent enormously larger than such molecules, which thus see an essentially planar wavefront with no chiral selectivity. It is conceivable that this situation will change under more specialized conditions, however, for example, in tight focusing or at shorter wavelengths.

## Footnotes

1 The prefix ‘pseudo’ signifies unusual transformation properties under a parity inversion. For example, a pseudoscalar changes sign under a parity inversion, whereas a scalar does not; a pseudovector does not change sign under a parity inversion, whereas a vector does [2].

2 Lipkin [60] does not elaborate upon his choice of the name ‘zilch’.

3 The quantity  vanishes for illuminating light made up of a single plane wave but is non-vanishing in general.
